# Clinical Frailty Scale as a Predictor of Early Treatment Discontinuation in Elderly Patients With Chronic Lymphocytic Leukemia Treated With Zanubrutinib: A Multicenter Real‐World Study

**DOI:** 10.1002/hon.70166

**Published:** 2025-12-29

**Authors:** Ernesto Vigna, Enrica Antonia Martino, Annalisa Pitino, Raffaella Pasquale, Isacco Ferrarini, Riccardo Moia, Andrea Visentin, Alessandro Sanna, Marina Motta, Massimo Moratti, Paolo Sportoletti, Annalisa Chiarenza, Alessandro Maggi, Valentina Zammit, Michele Merli, Idanna Innocenti, Claudia Giordano, Laura Nocilli, Massimiliano Postorino, Caterina Stelitano, Andrea Ferrario, Anna Maria Frustaci, Marcello Riva, Sara Pepe, Adalberto Ibatici, Stefania Scardino, Paola Anticoli Borza, Laura Ballotta, Salvatrice Mancuso, Francesco Malaspina, Anna Mele, Sara Galimberti, Gioacchino Catania, Annamaria Giordano, Ilaria Angeletti, Luana Schiattone, Elsa Pennese, Rosanna Miccolis, Angelo Fama, Giulio Giordano, Catello Califano, Antonella Bruzzese, Santino Caserta, Giuliana Farina, Pietro Bulian, Giacomo Loseto, Barbara Pocali, Vanessa Innao, Piero Galieni, Vincenzo Fraticelli, Candida Vitale, Azzurra Romeo, Marco Rossi, Ilaria Scortechini, Federico Vozella, Luigi Malandruccolo, Marzia Varettoni, Lucia Morello, Giuseppe Pietrantuono, Esmeralda Conte, Martina Cantelli, Roberta Murru, Daniele Caracciolo, Enrico Derenzini, Valentina Di Martina, Roberto Marasca, Maria Ilaria Del Principe, Amalia Figuera, Francesco Angotzi, Marta Coscia, Nicola Di Renzo, Luca Laurenti, Nicola Amodio, Pellegrino Musto, Francesco Di Raimondo, Arcangelo Liso, Alessandra Tedeschi, Livio Trentin, Gianluca Gaidano, Francesca Romana Mauro, Giovanni Tripepi, Andrea Corsonello, Fortunato Morabito, Valter Gattei, Massimo Gentile

**Affiliations:** ^1^ Department of Onco‐Hematology Hematology Unit Azienda Ospedaliera Annunziata Cosenza Italy; ^2^ Institute of Clinical Physiology (IFC‐CNR) Rome Italy; ^3^ Azienda Sanitaria Universitaria Friuli Centrale (ASU FC) SOC Clinica Ematologia Udine Italy; ^4^ Department of Engineering for Innovation Medicine University of Verona Verona Italy; ^5^ Division of Hematology Department of Translational Medicine Università del Piemonte Orientale Novara Italy; ^6^ Hematology Unit Department of Medicine‐DIMED University of Padova Padova Italy; ^7^ Hematology Unit Deparment of Oncology AOU‐Careggi Florence Italy; ^8^ Department of Hematology ASST Spedali Civili di Brescia Lombardia Italy; ^9^ Centro di Ricerca Emato‐Oncologica (CREO) Dipartimento di Medicina e Chirurgia Università di Perugia Perugia Italy; ^10^ Division of Hematology Azienda Policlinico‐S. Marco University of Catania Catania Italy; ^11^ Haematology Ospedale G. Moscati Taranto Italy; ^12^ Oncoematology and BMT Unit Oncology Department Ospedale La Maddalena Palermo Palermo Italy; ^13^ Hematology Fondazione IRCCS Ca’ Granda Ospedale Maggiore Policlinico Milano Italy; ^14^ Dipartimento di Scienze di Laboratorio ed Ematologiche Area Ematologia Fondazione Policlinico Universitario A. Gemelli IRCCS Università Cattolica del Sacro Cuore Rome Italy; ^15^ Department of Clinical Medicine and Surgery Federico II University Medical School Naples Italy; ^16^ Department of Hematology Azienda Ospedaliera Papardo Messina Italy; ^17^ Hematology Department of Biomedicine and Prevention Tor Vergata University of Rome Rome Italy; ^18^ Department of Hematology Azienda Ospedaliera Bianchi Melacrino Morelli Reggio Calabria Italy; ^19^ Department of Medicine and Surgery University of Insubria and Department of Oncology ASST Sette Laghi Ospedale di Circolo Varese Italy; ^20^ Department of Haematology Niguarda Cancer Center ASST Grande Ospedale Metropolitano Niguarda Milan Italy; ^21^ Hematology Department Ospedale San Bortolo di Vicenza Vicenza Italy; ^22^ Hematology Department of Translational and Precision Medicine Sapienza University Rome Italy; ^23^ Hematology and Cellular Therapy Unit IRCCS Ospedale Policlinico San Martino Genoa Italy; ^24^ Hematology and Stem Cell Transplant Unit “Vito Fazzi” Hospital Lecce Italy; ^25^ UOC Ematologia Azienda Ospedaliera San Giovanni‐Addolorata Rome Italy; ^26^ Dipartimento Clinico di Scienze Mediche Chirurgiche e della Salute Università degli Studi di Trieste Trieste Italy; ^27^ Department of Health Promotion Mother and Child Care Internal Medicine and Medical Specialties (PROMISE) Hematology Unit University of Palermo Palermo Italy; ^28^ Hematology Unit IRCCS IRST Istituto Scientifico Romagnolo per lo Studio e la Cura dei Tumori “Dino Amadori” Meldola Italy; ^29^ Haematology Ospedale Cardinale Panico Tricase(Lecce) Italy; ^30^ Division of Hematology University Hospital of Pisa Pisa Italy; ^31^ SCDU Ematologia ‐AOU SS Antonio e Biagio e C. Arrigo. Alessandria Italy; ^32^ Unit of Hematology and Stem Cell Transplantation AOUC Policlinico Bari Italy; ^33^ Department of Medicine and Surgery University of Perugia Hematology/Oncology Unit Terni Hospital Terni Italy; ^34^ Dipartimento di Ematologia Clinica Ospedale civile S. Spirito Pescara Pescara Italy; ^35^ Department of Hematology ASL BT Barletta Italy; ^36^ Department of Internal Medicine Hematology Unit Ospedale G. Mazzini Teramo Italy; ^37^ Unità Operativa Complessa Medicina Servizio e Ambulatorio di Ematologia Ospedale di Riferimento Regionale Antonio Cardarelli Campobasso Italy; ^38^ Onco‐Hematology Unit “A. Tortora” Hospital Pagani Italy; ^39^ Hematology Hospital “Sant'Anna e San Sebastiano” Caserta Italy; ^40^ Centro di Riferimento Oncologico di Aviano (CRO) Clinical and Experimental Onco‐Hematology Unit IRCCS Aviano Italy; ^41^ Hematology and Cell Therapy Unit IRCCS Istituto Tumori ‘Giovanni Paolo II’ Bari Italy; ^42^ Division of Hematology and Stem Cell Transplantation Unit Cardarelli Hospital Naples Italy; ^43^ Hematology Unit Azienda Ospedaliera di Rilievo Nazionale e di Alta Specializzazione (ARNAS) Garibaldi Catania Italy; ^44^ Department of Haematology and Stem Cell Transplantation Unit C. e G. Mazzoni Hospital Ascoli Piceno Italy; ^45^ Department of Hematology Gemelli Molise Campobasso Italy; ^46^ Division of Hematology A.O.U. Città della Salute e della Scienza di Torino Turin Italy; ^47^ Department of Molecular Biotechnology and Health Sciences University of Turin Turin Italy; ^48^ Hematology Unit Ospedale Santa Maria Goretti Latina Italy; ^49^ Department of Hematology‐Oncology Azienda Universitaria Ospedaliera Renato Dulbecco Catanzaro Italy; ^50^ Department of Experimental and Clinical Medicine Magna Grecia University Catanzaro Italy; ^51^ Azienda Ospedaliero Universitaria delle Marche Ancona Italy; ^52^ Hematology Stem cell Transplantation Fondazione Policlinico Universitario Campus Bio Medico di Roma Rome Italy; ^53^ Hematology Unit Ospedale “Fabrizio Spaziani” Frosinone Italy; ^54^ Division of Hematology Fondazione IRCCS Policlinico San Matteo di Pavia Pavia Italy; ^55^ Unità Operativa di Ematologia Ospedale Guglielmo da Saliceto Piacenza Italy; ^56^ IRCCS CROB Referral Cancer Center of Basilicata Rionero in Vulture Italy; ^57^ Haematology Unit Department of Clinical and Molecular Medicine Sant'Andrea University Hospital Sapienza University Rome Italy; ^58^ Unità di Ematologia Ospedale Santa Maria delle Croci Ravenna Italy; ^59^ Hematology and Stem Cell Transplantation Unit Ospedale Oncologico A. Businco ARNAS G. Brotzu Cagliari Italy; ^60^ Division of Onco‐Hematology IEO European Institute of Oncology IRCCS Milano Italy; ^61^ Hematology Unit Ospedale San Vincenzo Taormina Italy; ^62^ Hematology Unit Department of Oncology and Hematology Azienda‐Ospedaliero Universitaria (AOU) of Modena Policlinico Modena Modena Italy; ^63^ Department of Precision and Regenerative Medicine and Ionian Area “Aldo Moro” University School of Medicine Bari Italy; ^64^ Clinical Epidemiology and Physiopathology of Renal Diseases and Hypertension of Reggio Calabria Institute of Clinical Physiology (IFC‐CNR) Reggio Calabria Italy; ^65^ Unit of Geriatric Medicine Italian National Research Center on Aging (IRCCS INRCA) Cosenza Italy; ^66^ Department of Pharmacy Health and Nutritional Science University of Calabria Rende Italy; ^67^ AIL Sezione di Cosenza Cosenza Italy

**Keywords:** chronic lymphocytic leukemia, clinical frailty scale, frailty, treatment discontinuation, zanubrutinib

## Abstract

The management of chronic lymphocytic leukemia (CLL) in older patients requires careful balancing of therapeutic efficacy with the risks of treatment intolerance. Frailty assessment is increasingly recognized as a critical determinant of clinical outcomes, but its specific role in guiding therapy with second‐generation Bruton tyrosine kinase inhibitors remains poorly defined. We conducted a prospective, multicenter investigation of 326 consecutive CLL patients aged 65 years or older who received zanubrutinib across 52 Italian centers, aiming to evaluate whether the Clinical Frailty Scale (CFS) could predict treatment discontinuation in real‐world practice. The cohort was characterized by advanced age (median 78.1 years, range 65.1–94.5), with over half of the patients presenting with Binet stage C disease. Two‐thirds were treated in the frontline setting, while the remainder received zanubrutinib as salvage therapy. After a median follow‐up of 8 months, 48 patients (14.7%) discontinued treatment, most commonly due to toxicity or disease progression. Receiver operating characteristic curve analysis identified a CFS of 3 as the optimal threshold for predicting discontinuation, with an area under the curve of 0.65 (95% CI 0.56–0.73, *p* < 0.001). At 12 months, the discontinuation rate was significantly higher among patients with a CFS > 3 (29.2%) compared with those with a CFS ≤ 3 (8.8%) (*p* < 0.001); among conventional prognostic variables, only relapsed/refractory disease demonstrated an independent association with TTD. These findings highlight the CFS as a simple yet powerful clinical tool that provides incremental prognostic information beyond standard disease‐related factors. Incorporating frailty assessment into treatment planning may enhance patient selection and optimize therapeutic strategies for elderly CLL patients in daily practice.

## 1 Introduction

Chronic lymphocytic leukemia (CLL) is the most common hematologic malignancy in Western countries, primarily affecting older adults who are more susceptible to age‐related health declines [1].

At treatment initiation, many elderly patients with CLL already present with multiple chronic conditions, which can impair physical function and compromise adherence to therapy [1, 2].

Over the last decades, the introduction of targeted oral agents has markedly improved treatment outcomes in CLL, leading to durable remissions and prolonged survival [2]. However, these therapies also pose new challenges, particularly regarding long‐term adherence and the risk of drug–drug interactions in a comorbid population [3].

Frailty, defined as a state of increased vulnerability to stressors due to age‐related decline in physiological reserves and functions, has emerged as a key prognostic factor in oncology. In older cancer patients, frailty assessment helps identify individuals at higher risk for treatment‐related toxicity and early mortality [4]. The European Society for Medical Oncology (ESMO) recommends incorporating frailty assessment into treatment decision‐making, as it is strongly associated with impaired physical and cognitive performance as well as reduced survival in CLL [5]. Additional instruments such as the Cumulative Illness Rating Scale (CIRS) and the Charlson Comorbidity Index (CCI) are frequently applied in CLL to quantify comorbidity burden, which has been correlated with inferior overall survival. Nevertheless, comprehensive geriatric assessments remain underutilized in routine practice, largely due to their complexity, time requirements, and specialized expertise [6, 7, 8].

The prognostic role of frailty in elderly CLL patients has been prospectively evaluated in the CLL‐Frail trial, which assessed the FRAIL scale in patients aged ≥ 80 years or otherwise classified as frail and treated with acalabrutinib [9]. This study established the feasibility of integrating frailty assessment into BTKi therapy and provided benchmark data for treatment outcomes in this population. However, the trial was limited by a small sample size and relied on a patient‐reported measure rather than a clinician‐assessed scale, leaving open questions regarding the applicability of objective frailty tools in larger, real‐world cohorts.

Recently, our group investigated the use of the Clinical Frailty Scale (CFS) for frailty assessment in elderly CLL patients treated with novel targeted agents, including Bruton's tyrosine kinase inhibitors (BTKis) and BCL2 inhibitors (BCL2is) [10]. The CFS, originally developed within the Canadian Study of Health and Aging (CSHA), is a simple 9‐point scale that provides a rapid global assessment of an older adult's overall fitness or frailty, making it a practical tool in busy clinical settings [11].

The present study aims to evaluate the predictive value of the CFS for therapy discontinuation in a cohort of 326 consecutive elderly CLL patients, both treatment‐naive and relapsed‐refractory, treated with second‐generation BTKi, zanubrutinib.

## 2 Materials and Methods

### 2.1 Study Design and Population

This study encompasses a cohort of 326 consecutive patients aged over 65 years, affected by CLL and treated with zanubrutinib as first‐line or subsequent therapy between May 2024 and the time of data cutoff, across 52 Italian centers. All patients signed a written informed consent. Frailty was assessed by physicians using CFS. Comprehensive demographic and clinical data were collected, including age, sex, CIRS score, and concomitant cardiovascular risk factors. Disease‐related characteristics included Binet staging, TP53 aberrations (17p deletion and/or TP53 mutations), and IGHV gene mutational status. Adverse events leading to therapy discontinuation were registered according to the Common Terminology Criteria for Adverse Events (CTCAE) version 5.0. Discontinuations due to surgery were not considered treatment‐related toxicity. The study was approved by the Institutional Ethics Committees of all participating centers prior to data collection, in compliance with Italian regulations. The study was also conducted in accordance with the Declaration of Helsinki and Good Clinical Practice.

### 2.2 CSHA Clinical Frailty Scale

The CFS, developed within the CSHA [11], is a validated 9‐point tool that classifies older individuals according to functional capacity, vulnerability, and risk of mortality. The scale ranges from 1 (indicating robust health) to 9 (terminally ill) (Table 1). Frailty assessment was performed by trained physicians at treatment initiation, with each score assigned based on a physical examination and overall clinical impression. The scale ranges from 1 (very fit) to 9 (terminally ill) (Table 1). CFS assessments were conducted by trained hematologists at each participating center following CSHA guidelines. Although formal inter‐rater reliability was not evaluated, minor variability in scoring across centers cannot be excluded.

**TABLE 1:** The Canadian study of health and aging clinical frailty scale version 2.0^a^.

Level 1	Very fit: People who are robust, active, energetic, and motivated. These people commonly exercise regularly. They are among the fittest for their age.
Level 2	Fit: Previously known as well: People who have no intense disease symptoms but are less fit than level 1. Often, they exercise or are very active occasionally, for example, seasonally.
Level 3	Managing well: People whose medical problems are well controlled, but are not regularly active beyond routine walking.
Level 4	Living with very mild frailty‐while not dependent on others for daily help, often symptoms limit activities. A common complaint is being “slowed‐up” and being tired during the day.
Level 5	Living with mild frailty: These people usually have more evident slowing and need help in higher‐order instrumental activities of daily living (IADLs) such as finance, transportation, heavy housework, medications. Typically, mild frailty progressively impairs shopping and walking outside alone, meal preparation, and housekeeping.
Level 6	Living with moderate frailty: They need help with all outside activities and with keeping house. Inside, they often have problems with stairs and need help with bathing, and might need minimal assistance (standby) with dressing.
Level 7	Living with severe frailty: is characterized by progressive dependence in personal ADLs. Completely dependent on personal care from whatever cause (physical or cognitive). Even though they seem stable and not at high risk of dying (within 6 months).
Level 8	Living with very severe frailty: These patients are completely dependent, approaching the end of life. Typically, they could not recover even from minor illnesses.
Level 9	Terminally Ill: Approaching the end of life. This category applies to people with a life expectancy of under 6 months, which are not otherwise evidently frail.

^a^ Rockwood K, Theou O. Using the clinical frailty scale in allocating scarce health care resources. Canadian Geriatrics Journal. 2020 Sep; 23(3):210.

### 2.3 Analytic Approach

The primary objective of the study was to evaluate the prognostic role of CFS in predicting time to discontinuation (TTD).

TTD was defined as the interval from treatment initiation to permanent discontinuation for any cause, including adverse events, disease progression, transformation to Richter's syndrome, or death, with a treatment interruption of > 30 days considered a discontinuation event. Competing risks, such as death, were not formally modeled in this analysis. All adverse events resulting in definitive treatment discontinuation were graded > 3 and considered irreversible. Only grade ≥ 3 adverse events leading to treatment discontinuation were considered; lower‐grade events potentially affecting adherence were not captured. Patients who were still receiving zanubrutinib at data cutoff were censored at their last follow‐up.

All included patients received zanubrutinib at the standard dose of 320 mg daily. Categorical variables were analyzed using Fisher's exact test (for two‐way tables) and Pearson's χ^2^ test (for multi‐way tables). Associations between individual variables and overall survival (OS) were assessed with the log‐rank test, and results were presented as hazard ratios (HRs) with 95% confidence intervals (CIs). The predictive performance of the CFS was evaluated using receiver operating characteristic (ROC) curve analysis, calculating the area under the curve (AUC), along with sensitivity, specificity, and both positive and negative predictive values, all reported with 95% CIs. The optimal cut‐off point for CFS was identified using Youden's index (*J* = sensitivity + specificity − 1), with the maximum value of *J* (0.243) indicating the threshold that best balanced sensitivity and specificity. The prognostic relevance of each variable was first assessed through univariable Cox regression analysis, with HRs and 95% CIs reported. Variables found to be statistically significant (*P* < 0.05) in univariable analysis were then included in a multivariable Cox regression model. All statistical analyses were conducted using STATA for Windows version 9 and SPSS Statistics version 21.

## 3 Results

### 3.1 Patients

Baseline characteristics of the study cohort are summarized in Table 2. The median age at zanubrutinib initiation was 78.1 years (range 65.1–94.5 years). More than half of the cohort (52.1%) presented with Binet stage C disease, while the remaining patients (stage A/B) initiated treatment due to disease progression or disease‐related symptoms, according to the International Workshop on CLL guidelines [8].

**TABLE 2:** Baseline characteristics of the CLL patients treated with zanubrutinib.

	Zanubrutinib (*n* = 326)
Median age, years (range)	78,1 (65,1–94,5)
Sex, *n* (%)	
Male	195 (59.8)
Female	131 (40.2)
Line of therapy, *n* (%)^a^	
TN	208 (63.8)
R/R	118 (36.2)
Binet stage, *n* (%)	
A	2 (9.8)
B	124 (38)
C	170 (52.1)
IGHV mutational status, *n* (%)	
Mutated	97 (29.8)
Germline	190 (58.3)
Unknown	39 (12)
TP53 disruption, *n* (%)^b^	
Absent	218 (66.9)
Present	68 (20.8)
Unknown	40 (12.3)
CLL‐IPI risk (%)	
Low	13 (4)
Intermediate	67 (20.5)
High	122 (37.4)
Very‐high	59 (18.1)
Unknown	65 (20)
CIRS *n* (%)	
0–6	198 (60.7)
> 6	128 (39.3)
Clinical frailty scale *n* (%)	
1	46 (14.1)
2	74 (22.7)
3	108 (33.1)
4	53 (16.3)
5	25 (7.7)
6	11 (3.4)
7	9 (2.8)
Cardiovascular risk factor, *n* (%)	
Hypertension	139 (42.6)
Atrial fibrillation	49 (15)
Hypercholesterolemia	54 (16.6)
Congestive heart failure	24 (7.4)
Peripheral arterial disease	12 (3.7)
Cerebrovascular disease	13 (4)
Diabetes	62 (19)
Myocardial infarction	47 (14.4)

^a^ TN, treatment naïve; R/R, refractory/relapsed.

^b^ TP53 disruption was determined by the documentation of 17p deletion and/or TP53 mutation by FISH and sequencing, respectively; present = del17p positive and/or TP53 mutated; absent = del17p negative and TP53 negative.

With regard to IGHV gene status, 29.8% of patients were mutated, 58.3% unmutated, and in 11.9% the information was unavailable. Cytogenetic analysis revealed del(17p) and/or TP53 mutations in 20.8% of patients (68 cases). A CIRS score > 6 at therapy initiation was observed in over one‐third of the cohort. Hypertension was the most frequent comorbidity (42.6%). Atrial fibrillation and previous myocardial infarction were reported in 15% and 14.4% of patients, respectively. Additional cardiovascular risk factors included diabetes (19%) and hypercholesterolemia (16.6%). According to the CSHA CFS, 46 patients (14.1%) scored 1, 74 (22.7%) scored 2, 108 (33.1%) scored 3, 53 (16.3%) scored 4, 25 (7.7%) scored 5, 11 (3.4%) scored 6, and 9 (2.8%) scored 7; no cases scored 8–9. Overall, 63.8% (208 cases) were treatment‐naïve, while 36.2% (118 patients) received zanubrutinib as salvage therapy. In the subgroup of the treatment‐naïve, 28 patients (13.5%) showed a CSHA CFS of 1, 45 (21.6%) of 2, 73 (35.1%) of 3, 31 (14.9%) of 4, 18 (8.7%) of 5, 8 (3.8%) of 6, and 5 (2.4%) of 7; in the subgroup of relapsed/refractory 18 patients (15.3%) showed a CSHA CFS of 1, 29 (24.6%) of 2, 35 (29.7%) of 3, 22 (18.6%) of 4, 7 (5.9%) of 5, 3 (2.5%) of 6, and 4 (3.4%) of 7.

### 3.2 Safety

After a median follow‐up of 13 months, treatment was permanently discontinued in 48 patients (18.7%). The major causes for discontinuation were treatment‐related toxicity and disease progression (Table 3). Specifically, 14 patients (5.8%) discontinued due to adverse events: 6 cases for infections, 5 for bleeding, 1 for atrial fibrillation, 1 for rash, and 1 for HBV reactivation. Disease progression accounted for 17 discontinuations, including 1 Richter's transformation. An additional 13 patients (1.5%) discontinued treatment due to deaths (6 cases unrelated to CLL and 7 for unknown causes) (Table 3). Three patients discontinued treatment due to the occurrence of a second malignancy, and 1 patient withdrew consent.

**TABLE 3:** Reasons for zanubrutinib discontinuation in patients with CLL.

	Zanubrutinib
Total patients	326
Patients who discontinued treatment, *n* (%)	48 (18.7)
Median follow‐up (months)	13
Reasons for discontinuation	
Toxic effect of therapy	14 (4.3)
Infection	6 (1.8)
Bleeding episodes	5 (1.5)
Atrial fibrillation	1 (0.3)
Rash	1 (0.3)
Hepatitis B virus reactivation	1 (0.3)
Disease progression	
CLL	16 (4.9)
Richter syndrome	1 (0.3)
Second neoplasia	3 (0.9)
Patient request	1 (0.3)
not‐CLL related death	6 (1.8)
Death from unknown causes	7 (2.1)

### 3.3 CSHA CFS and Prediction of Treatment to Discontinuation

**FIGURE 1:**
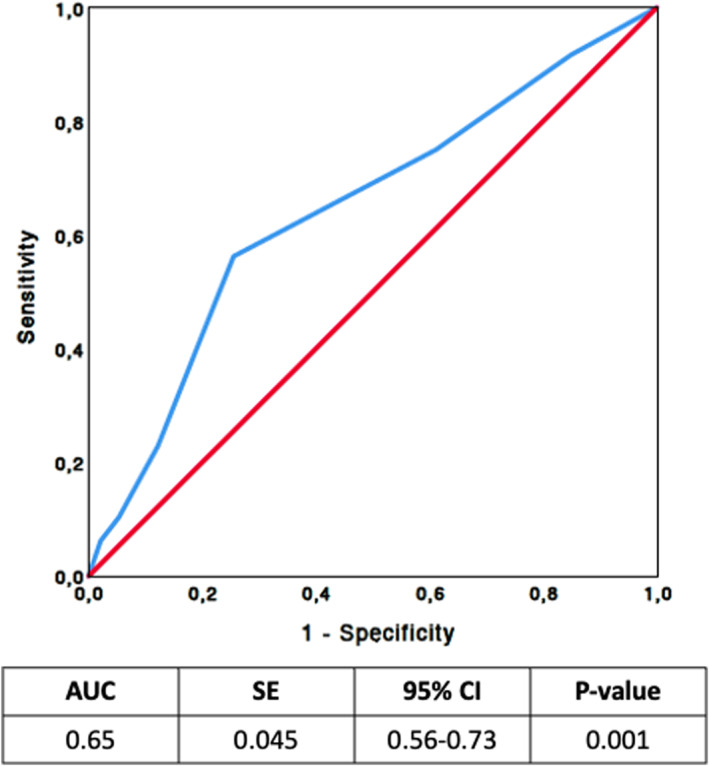
Receiver operating characteristic (ROC) analysis of canadian study of health and aging (CSHA) clinical frailty scale (CFS) for predicting time to discontinuation (TTD) of CLL patients treated with zanubrutinib.

**FIGURE 2:**
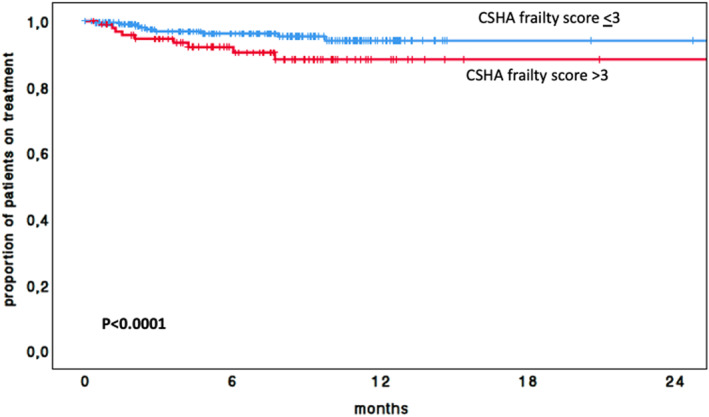
Kaplan‐meier curve of TTD of the entire cohort according to the canadian study of health and aging (CSHA) clinical frailty scale (CFS).

The predictive value of the CSHA CFS for TTD was evaluated using ROC curve analysis (Figure 1). The AUC was 0.65 (95% CI: 0.56–0.73; *p* < 0.001). The optimal cutoff point was identified at a CFS of 3, corresponding to a sensitivity of 56% and specificity of 75%. Overall, 228 (69.9%) had a CFS ≤ 3, while 98 (30.1%) had a CFS > 3. At 12 months, the discontinuation rate was significantly higher among patients with a CFS > 3 (29.2%) compared with those with a CFS ≤ 3 (8.8%) (*p* < 0.001; Figure 2 and Table 4).

**TABLE 4:** Univariate and multivariate analyses for time to discontinuation (TTD).

	Univariate analysis		Multivariate analysis	
	HR (95% CI)	*p* value	HR (95% CI)	*p* value
Age^a^	1.052 (1.007–1.098)	**0.021**	1.02 (0.98–1.07)	0.33
Sex				
Male versus female	0.88 (0.49–1.59)	0.68	—	—
Line of therapy^b^				
R/R versus TN	2.03 (1.15–3.59)	**0.015**	1.92 (1.08–3.41)	**0.027**
Binet stage				
B versus A	1.83 (0.53–6.36)	0.34	—	—
C versus A	1.69 (0.5–5.67)	0.4	—	—
IGHV mutational status				
Germline versus mutated	1.66 (0.81–3.39)	0.17	—	—
TP53 disruption^c^				
Present versus absent	1.47 (0.74–2.93)	0.27	—	—
Clinical frailty scale
≤ 3 versus > 3	3.3 (1.87–5.84)	**<** **0.0001**	2.74 (1.47–5.09)	**0.001**
CIRS				
< 6 versus ≥ 6	1.33 (0.75–2.36)	0.32		
Cardiovascular risk factor				
Hypertension	1.08 (0.61–1.91)	0.9	—	—
Atrial fibrillation	1.1 (0.49–2.45)	0.65	—	—
Hypercholesterolemia	2.2 (0.79–6.16)	0.13	—	—
Congestive heart failure	3.07 (1.43–6.58)	**0.004**	2.14 (0.98–4.68)	0.06
Peripheral arterial disease	1.07 (0.25–4.48)	0.93	—	—
Cerebrovascular disease	1.13 (0.27–4.67)	0.86	—	—
Diabetes	1.57 (0.67–3.71)	0.29	—	—
Myocardial infarction	1.38 (0.67–2.85)	0.39	—	—
≥ 1 cardiovascular risk factor	1.02 (0.77–1.35)	0.89	—	—

*Note:* The bold values indicate statistically significant.

^a^ The effect corresponds to 1 year of increase in age.

^b^ R/R, refractory/relapsed; TN, treatment naïve.

^c^ TP53 disruption was determined by the documentation of 17p deletion and/or TP53 mutation by FISH and sequencing, respectively; present = del17p positive and/or TP53 mutated; absent = del17p negative and TP53 negative.

At univariate analysis, in addition to a CFS > 3, age, treatment setting (treatment‐naïve vs. relapsed/refractory) and congestive heart failure showed a significant association with treatment discontinuation. At multivariate analysis only CFS > 3 (HR = 1.92, 95% CI: 1.08–3.41; *p* = 0.001) and relapsed/refractory disease (HR = 2.74, 95% CI: 1.47–5.09; *p* = 0.027) remained statistically associated with a shorter TTD (Table 4).

## 4 Discussion

Geriatric assessment provides a comprehensive evaluation of elderly patients' health status, enabling identification of those at increased risk of treatment‐related toxicity and early therapy discontinuation. Integrating geriatric dimensions into routine clinical practice remains highly relevant, even in the era of targeted therapies.

Several clinical trials [12, 13] have highlighted the prognostic impact of geriatric domains—including functional, psychological, and cognitive status, as well as social support—on survival, underscoring the importance of incorporating the frailty evaluation into therapeutic decision‐making for CLL patients. Such assessment may help identify individuals more likely to experience toxicities and guide the implementation of supportive measures.

In our previous study [10] of 82 CLL patients aged ≥ 65 years treated with BTKis or BCL‐2is, the CSHA CFS proved to be a simple and effective tool for distinguishing patients able to tolerate small‐molecule therapy from those who were not. ROC curve analysis identified a value of 3, above which patients were more likely to discontinue therapy prematurely.

The CLL‐Frail trial represents the first prospective study evaluating frailty in elderly CLL patients treated with a BTK inhibitor, using the patient‐reported FRAIL scale to identify individuals at higher risk of treatment‐related toxicity and adverse outcomes [9]. This study demonstrated the feasibility of integrating frailty assessment into treatment planning and provided important benchmark data for therapy response and early discontinuation. Our study complements these findings by assessing frailty using a clinician‐judged global measure (CFS) in a larger, multicenter, real‐world cohort treated with zanubrutinib. Unlike the FRAIL scale, which relies on patient‐reported functional items, the CFS provides a rapid, objective bedside evaluation of overall physiological reserve and vulnerability, allowing a practical assessment that can inform therapy decisions and predict early discontinuation in routine practice. However, direct head‐to‐head comparisons are lacking and warranted in future studies.

The present analysis, conducted in a larger cohort of elderly CLL patients treated exclusively with zanubrutinib, confirmed the prognostic validity of this cut‐off. Patients who scored ≥ 3 had nearly a 2.7‐fold likelihood of early treatment interruption compared with those scoring ≤ 3. These findings support the hypothesis that dose adjustment strategies could be beneficial for frail patients deemed unlikely to tolerate full‐dose therapy. While pharmacokinetic and pharmacodynamic data specifically for zanubrutinib in frail patients are not available, studies with other BTK inhibitors, such as ibrutinib have demonstrated that full‐dose administration produces drug concentrations exceeding those required for a complete occupancy of the BTK receptor [14]. These observations suggest that, in frail patients at higher risk of early discontinuation, careful dose adjustment strategies could potentially improve adherence and prolong treatment exposure, though this hypothesis requires formal evaluation in prospective studies.

Although no clinical trial has demonstrated that educed doses of BTK inhibitors achieve clinical outcomes comparable to standard dosing in terms of response rates, PFS, or OS compared and zanubrutinib dose reductions have generally been associated with inferior outcomes in general [15], it is conceivable that in highly frail patients at increased risk of early discontinuation, carefully selected dose modifications reduced dosing could improve tolerability, enhance adherence, and ultimately prolong treatment exposure. This hypothesis remains speculative and warrants prospective evaluation. Relapsed/refractory disease, together with a CSHA CFS > 3, was independently associated with treatment discontinuation. This is expected, as relapsed/refractory patients have a higher incidence of disease progression compared with first‐line patients (7% vs. 3%).

Interestingly, other clinical and biological parameters—including age, gender, CIRS > 6, Binet stage, IGHV mutational status, and TP53 abnormalities—did not significantly influence the risk of treatment discontinuation in our study.

With a median follow‐up of 13 months, 14 out of 326 patients (4.3%) discontinued zanubrutinib due to toxicity. To note, limited cardiac events were recorded (1 case of atrial fibrillation), and among the off‐target effects, 5 bleeding events led to drug withdrawal. Although the short follow‐up limits definitive conclusions, these findings reinforce the favorable tolerability profile of zanubrutinib and support its feasibility in real‐world use among older CLL patients. A recent real‐world study from Kaiser Permanente Northern California [16], including 281 CLL patients treated with ibrutinib followed by zanubrutinib or zanubrutinib alone, found that in the zanubrutinib‐only cohort—where at least half of the patients were ≥ 71 and had multiple comorbidities—after a median follow up of 8.2 months, zanubrutinib was associated with low cardiotoxicity and a reduced rate of discontinuations due to adverse events.

Nonetheless, several important challenges remain regarding frailty assessment in CLL. First, most evidence on the prognostic value of geriatric tools derives from the chemoimmunotherapy era, with relatively few studies exploring their relevance in the context of targeted therapies. Second, the predictive value of frailty may be influenced by disease biology, particularly in cases with Richter's transformation or B symptoms. In our cohort, only one transformation to aggressive lymphoma occurred, limiting further interpretation. Third, the practical implementation of frailty assessments in daily practice is hampered by their complexity and the time required, particularly in high‐volume hematology clinics.

In conclusion, this real‐world multicenter study highlights the value of the CFS in assessing elderly patients with CLL treated with zanubrutinib.

Strengths include the pragmatic design, the inclusion of a frail population rarely represented in clinical trials, and the use of a simple bedside tool easily applicable in routine practice. CFS provides meaningful information beyond conventional prognostic markers and may help personalize therapy in the era of second‐generation BTK inhibitors. Compared with the FRAIL score, CFS is a clinician‐judged global frailty scale rather than a patient‐reported screening tool, offering a more nuanced and objective assessment, though direct comparisons are warranted.

The study also presents limitations. The relatively short follow‐up and the low number of discontinuation events limit the power of multivariable analyses and may increase the risk of overfitting. The definition of treatment discontinuation as *a* > 30‐day interruption, while consistent with real‐world practice, may overestimate events relative to standard iwCLL criteria. CFS assessments were physician‐judged across multiple centers without formal inter‐rater reliability testing, introducing potential variability. Competing risks, such as death, were not formally modeled. Only grade ≥ 3 adverse events resulting in permanent discontinuation were captured, potentially underestimating the impact of lower‐grade toxicities on adherence. Moreover, discussion of dose reductions remains speculative due to limited pharmacokinetic data for zanubrutinib in frail populations, underscoring the need for prospective validation. Finally, long‐term outcome evaluation, and the absence of direct comparison with other geriatric tools precludes definitive conclusions on its superiority.

Overall, CFS emerges as a feasible and informative approach to frailty assessment, warranting confirmation in larger, prospective studies with extended follow‐up.

## Author Contributions

All Authors contributed equally to this study.

## Funding

This study was supported by the PNRR‐MAD‐2022‐12375673.

## Ethics Statement

All participating centers obtained ethics approval for this study, which was conducted in accordance with the Declaration of Helsinki.

## Consent

Written informed consent has been obtained from the involved patients

## Conflicts of Interest

The authors declare no conflicts of interest.

## Data Availability

The data that support the findings of this study are available on request from the corresponding author. The data are not publicly available due to privacy or ethical restrictions.
